# Supplementary material for: Influence of augmented humans in online interactions during voting events
Source: PLoS One. 2019 May 16;14(5):e0214210. doi: 10.1371/journal.pone.0214210 (PMC6521989; doi:10.1371/journal.pone.0214210)
Supplement: S1 Text — (PDF) [file pone.0214210.s001.pdf]

Supplementary Information for Influence of augmented humans in online interactions during voting events

Massimo Stella[1,#a], Marco Cristoforetti[1], Manlio De Domenico[1*]

[1] Fondazione Bruno Kessler, Via Sommarive 18, 38123 Povo (TN), Italy
[#a] Current Address: *Complex Science Consulting*, Via A. Foscarini 2, 73199 Lecce (LE), Italy

[*] mdedomenico@fbk.eu

1 Bot detection

In this work the classification of users in our data set as "humans" or "bots" is based on features providing the best classification accuracy according to recent studies [?]: 1) *Statuses count*; 2) *Followers count*; 3) *Friends count*; 4) *Favourites count*; 5) *Listed count*; 6) *Default profile*; 7) *Geo enabled*; 8) *Profile use background image*; 9) *Protected*; 10) *Verified* for a total of ten features ($N_{feats} = 10$).

Searching for better performance we tested different machine learning techniques on an independent dataset created ad-hoc.

In particular for the models we considered: logistic regression (LOGR), ada-boost classfier (AB), random forest (RNF), stochastic gradient descent (SGD), deep learning neural network (DL). Apart from the deep learning architecture, for all the other models we use the implementation of the algorithms of the `scikit-learn` (http://scikit-learn.org/). The Deep Learning architecture instead was developed using the `pytorch` (http://pytorch.org/) framework and consist of four fully-connected layers of $2 \times N_{feats}$, $4 \times N_{feats}$, N_{feats} and 2 hidden nodes respectively. The activations are ReLU for all the layers except the for the last one that uses a Sigmoid, a dropout of 0.2 was also applied between the fully-connected layers in order to prevent overfitting.

1.1 Testing on independent data sets

From the side of the training and validation dataset we added to the public available datasets [?, ?] other lists of humans and bot we could find from different sources (see Tab **??**), in this way we increased the variety of bots and the size of the dataset with information on 22,993 users: 14,218 bots and 8,775 humans. In other words, we used all the user information from all the datasets reported in SI Table I, with 62% of bots and 38% of human users, in order to develop and train our machine learning algorithms through cross-validation. We used 80% of the dataset for training and 20% for validation, the subdivision between the two sets has been carried respecting the balancing between bots and humans present at the level of the single original datasets, in this way we have all type of different bots both in training and validation.

To fix the parameters of the models we perform the fit using three-fold cross validation on the training dataset. After this optimization procedures the models are tested on the validation data.

data set	bot	human	total
cresci2015	0	5301	5301
cresci2017	7543	3474	11017
cyborgs	2756	0	2756
aboutme	0	2463	2463
omnibots	3530	0	3530
russian-trolls	389	0	389
TOTAL	14218	8775	22993

Table 1: **Proportions of bot and human users in the training data.**

Different statistics were considered, to compare the performance of each model, as shown in Fig. **??**. From the figure emerges that random forest (RNF) and Deep Learning (DL), perform better than the other models in almost every metric. For this reason we test these two models on the Italian Election dataset. We know that in the dataset used for training the model there is a particular class of users that must be considered as bot in our analysis but are not present in the training dataset, these are mainstream broadcasters with a staff of journalists rather than single users behind them. For this reason we look at the performance of the two models on this particular class of bot and in particular on a list of nine bots that are present in our dataset: "you_trend", "EuropeElects", "matteosalvinimi", "repubblica", "Agenzia_Ansa", "TgLa7", "matteorenzi", "SkyTG24", "fattoquotidiano". Because in the Italian data set some users are borderline, and their "botness" can only be approximated with small confidence, we decided to collect statistics from 10 different round of training for the DL and RF models: different seed initializations will reflects in small differences in the accuracy on the training but can move the prediction on the borderline users in the Italian dataset. At every the end of the training we look at the stability of the prediction of the broadcasters as bot. This test showed that DL is more stable in the classification outcome. RF on the other hand often misses "you_trend" or "TgLa7". After this we chose DL as model for our analysis.

2 Evolution of social interactions across time

In order to gain insight about the evolution of social dynamics between humans and bots, their interactions are aggregated according to different temporal periods: (i) *Before* (from February 27 until March 2), (ii) *Eve* (March 3, the day before the referendum), (iii) *Voting day* (March 4) and (iv) *Aftermath* (from March 5 until March 6). A directed complex network, representing the observed social interactions (links) between users (nodes), is built for each of these time windows.

We start by analyzing homophily among humans and bots by the excess probability:

$$\Delta_{kl} = \frac{p_{kl} - p_{kl}^{(ran)}}{p_{kl}}, \qquad (1)$$

quantifying how much larger is the probability p_{kl} of finding a link from a user of a class k to a user of class l ($k, l =$ human, bot) compared to the case of a null model where social interactions are randomized. Since the number of social interactions (i.e., the degree) of a given user is an important

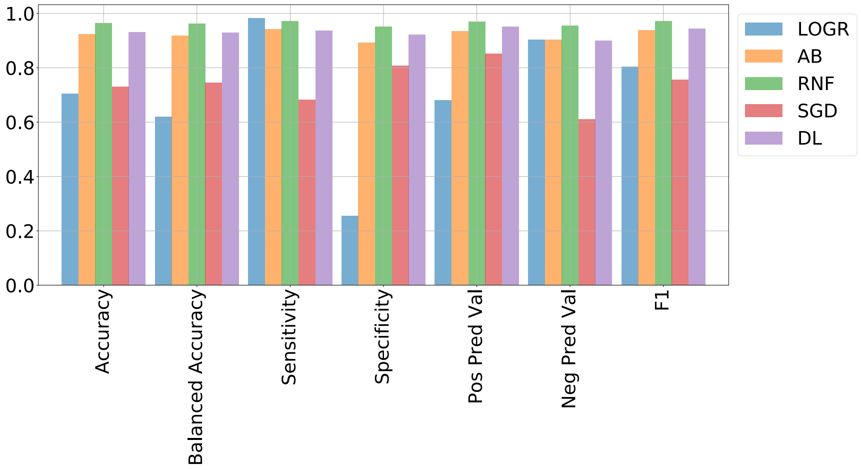

Figure 1: **Validation of different classification models.** We have tested different models against a validation data set. All models have been trained on the same data.

	Interaction Probability			
Time	$H \rightarrow H$	$B \rightarrow B$	$H \rightarrow B$	$B \rightarrow H$
Before	+4.3%	+20.7%	-3.4%	-56.1%
Eve	+3.2%	+16.4%	-2.3%	-39.8%
Voting	+3.7%	+16.8%	-2.7%	-42.6%
Aftermath	+5.2%	+7.4%	-1.0%	-20.8%

Table 2: **Bot-human assortative mixing.** Excess probability of observing an interaction between or within user categories – i.e., humans (H) and bots (B) – appropriately normalized by its random expectation (see Eq. (**??**)). Results highlight that humans tend to interact with bots less than at random (disassortative mixing) while bots tends to interact with other bots more than random expectation (assortative mixing). Assortative mixing of bots is present across the whole period of observation.

estimator of the influence of user itself in online social networks [**?**, **?**], we consider a null model fixing users' degree while randomizing their connections, also known as configuration model [**?**, **?**]. If $\Delta_{kl} > 0$ then interactions from class k to class l are over-represented in the observed social system, compared to the null model; $\Delta_{kl} < 0$ indicates instead that interactions are under-represented. The measure defined by Eq. (**??**) is not affected by size effects, because sizes are appropriately renormalized by the ratio random expectation. Table **??** reports excess probability for all possible human-bot interactions. During the whole period, bot-bot interactions are more likely than random ($\Delta_{BB} > 0$), indicating that bots tend to interact more with other bots rather than with humans ($\Delta_{BH} < 0$) during Italian elections. Since interactions often encode the spread of a given content online [**?**], the positive assortativity highlights that bots share contents mainly with each other and hence can resonate with the same content, be it news or spam.

3 Bots interactions are persistently targeted

It is natural to wonder if there is any relationship between the humans' centrality in the online social network and humans who are the preferred target of bots targeted content. To address this question we quantify the incoming degree, shortly indegree, of human users in the system. The indegree here corresponds to the aggregated number of received interactions and it is a good proxy of a user's influence in online social networks [?, ?]. To test the hypothesis that bots mostly target highly influential users, as recently found during other voting events [?], we calculate, by means of Kendall rank correlation coefficient τ, the correlation between the number of incoming interactions from humans and from bots. We find $\tau = 0.14$ (Before), $\tau = 0.14$ (Eve), $\tau = 0.07$ (Vote), and $\tau = 0.11$ (Aftermath), with p-values smaller than 10^{-3}. This results highlights a tendency, consistently observed over time, for bots to target the most influential human users in the considered online microblogging platform, confirming previous findings [?].

4 Influence of bots and humans over time

Quantifying the evolution in humans' and bots' influence over time is useful to understand if there are dramatic changes in the structure of the network or in the social dynamics. Here, we calculate three widely used centrality measures, namely (i) the number of users addressing a given user in their interactions (indegree), (ii) the number of users targeted by a given user with its interactions (outdegree), and (iii) the probability of finding a given user by exploring the web of social interactions at random (PageRank [?]). Since the size of the network changes over time in the four periods considered, a direct comparison of individuals' influence between different time windows might be affected by size effects. Instead, it is more interesting to quantify how the difference between humans and bots – calculated within each period, separately – evolves over time.

Results reported in Fig. ?? show that, during all periods, bots are almost twice as central as humans in terms of PageRank. This effect is even larger when indegree centrality is considered. No large differences between humans and bots are present in terms of outdegree. Because of PageRank and indegree, the most influential users across the whole period of Italian elections are bots.

Ranking users with the highest number of social interactions provides additional information on the nature of bots in the considered social system. Table ?? (top) ranks the first 10 social hubs in each time window. A total of 40% of hubs is represented by human users, with two of them being public profiles of Italian political leaders. The remaining six hubs are news media, which are categorized as (news) bots. This result is not surprising: news media profiles are managed by editorial staffs and hence produce content in a different way, if compared to the majority of users with personal accounts in online social platforms. The presence of news media and news organizations acting as broadcasters has already been detected in previous investigations [?].

Table ?? (top) highlights a large variability in terms of ranking across time periods, with some users playing the role of hubs during the voting day which lose even thousands of positions in their ranking either before or after the voting. These fluctuations can be explained by the high variance

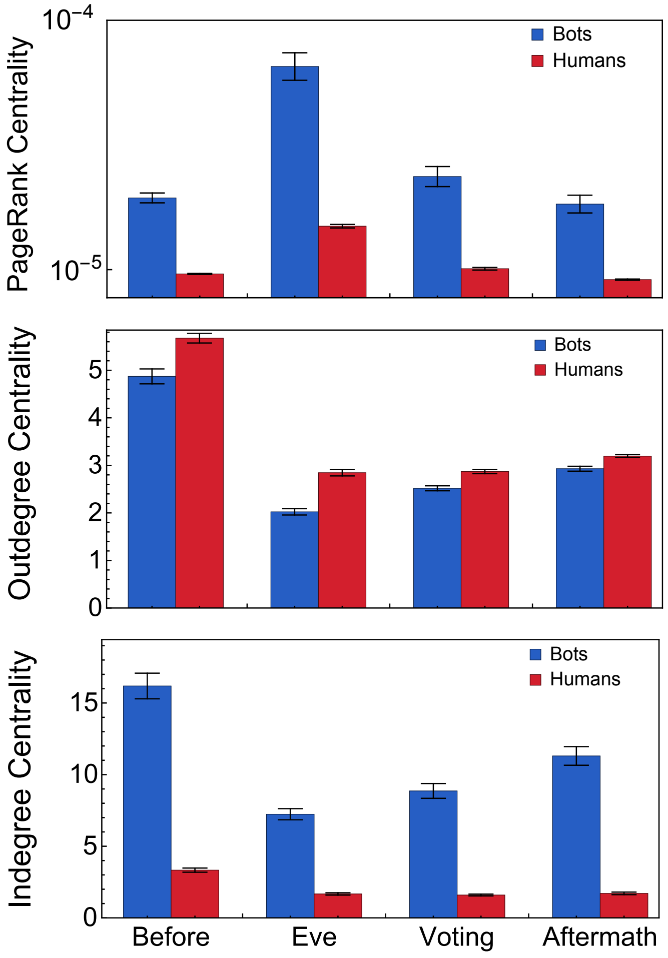

Figure 2: **Bots are the most central users over time.** Mean PageRank (top), outdegree (middle) and indegree (bottom) centralities for bots and humans during the four periods considered. Both the PageRank and indegree indicate that bots are more central than humans in terms of information flow consistently over time.

of the number of social interactions per day, which can highly vary from day to day for an individual user [**?**, **?**].

Table **??** (bottom) ranks hubs with less than 10^4 followers, to exclude organizations, political leaders and news media and highlight the role of more common users. In fact, the top users satisfying this criterion are humans. On average, rank fluctuations for individual users are larger than for broadcasters by at least one order of magnitude. These fluctuations for individual users indicate that ranking nodes according to the number of social interactions they are involved in might not be a good proxy for determining the most influential users in the system. This is why we need to: (i) reduce fluctuations by filtering out weak social interactions and (ii) identify better measures of influence for users in online social networks.

Top10 Voting	Rank Before	Rank - Eve	Rank - Voting	Rank - Aftermath	Mean Rank	Followers
**you_trend**	470	(+321) 149	(+148) 1	(-4) 5	150 ± 100	25K
**EuropeElects**	1027	(+786) 241	(+239) 2	(-4) 6	320 ± 240	45K
**ViolenzaDentro**	18	(+12) 6	(+3) 3	(-2601) 2604	660 ± 640	1.5K
**matteosalvinimi**	3	(-11) 14	(+10) 4	4	6 ± 3	661K
**repubblica**	10	(-1) 11	(+6) 5	(+2) 3	7 ± 2	2830K
**Agenzia_Ansa**	28	(+5) 23	(+17) 6	(-9) 15	18 ± 5	974K
**TgLa7**	264	(-76) 340	(+333) 7	(-9) 16	160 ± 80	544K
**matteorenzi**	2	(+1) 1	(-7) 8	(+7) 1	3 ± 2	3380K
**SkyTG24**	20	(-24) 44	(+35) 9	9	20 ± 8	3000K
**fattoquotidiano**	15	(+8) 7	(-3) 10	(-19) 29	15 ± 5	1900K

Top10 Voting	Rank Before	Rank - Eve	Rank - Voting	Rank - Aftermath	Mean Rank	Followers
**ViolenzaDentro**	18	(+12) 6	(+3) 3	(-2601) 2604	660 ± 640	1.5K
**Utente05**	571	(-13621) 14192	(+14179) 13	(-36) 49	3700 ± 3500	8K
**Utente06**	373	(-2098) 2471	(+2457) 14	(-730) 744	900 ± 500	6K
**Utente01**	-	2814	(+2798) 16	(+14) 2	944 ± 900	800
**Utente04**	14	(+12) 2	(-18) 20	(-777) 797	210 ± 190	7K
**Utente07**	40	(+36) 4	(-25) 29	(-5673) 5702	1440 ± 1420	163
**Utente08**	-	-	32	(-1331) 1373	702 ± 500	2K
**Utente09**	2	(-15205) 1	(+17089) 8	(-232) 1	3 ± 2	4K
**Utente10**	20	(+2218) 44	(+112) 9	(-313) 9	20 ± 8	6K
**Utente11**	4929	(-3153) 8082	(+8037) 45	(-1628) 1673	3700 ± 1800	2K

Table 3: **Top influencers are mostly bots.** Hubs characterize influential users and broadcasters in online social systems [**?**], hence we use degree rankings for identifying the most influential users in the network. Bot (blue) and human (red) users are ranked across the whole voting period. The difference in ranking between consecutive periods is reported between parentheses. The second last column indicates the mean rank with standard deviations to quantify rank variability across time. The last column indicates the size of the social neighborhood (followers). Top table: The 10 largest hubs during the whole voting period. Bottom table: The 10 largest hubs with less than 10,000 (10K) followers.

5 Semantic content of bot and human messages

We analyze the semantic content of messages exchanged by either bots or human users by considering hashtag associations, i.e. linking together in a network any two hashtags co-occurring in the same message coming from either a bot or a human. Results are reported in Fig. **??**.

Differently from previous works, where the semantic content of bots and humans differs in its emotional polarity [**?**], in here we find that bots mainly repeat the same political content of human users, thus boosting the spreading of hashtags strongly related to the electoral process, such as hashtags referring to the government or to political victory, names of political parties or names of influential politicians (see also **??**).

In order to better characterize this pattern, we computed: (i) the frequency of hashtags in messages shared by humans and bots and (ii) the closeness centrality of hashtags in networks of hashtag co-occurrence in the

same message. The frequency analysis reveals that in messages involving at least one bot the hashtag *#ultimora* was more than 34 times more frequent (i.e. x34) than in messages between humans only. Bots boosted also the frequency of hashtags such as *#primoposto* (x26), *#algoverno* (x5) and *#riforme* (x3). All these hashtags represent evidence that bots boosted neutral political content related to the voting process.

Frequencies of individual hashtags during the whole electoral process display some interesting shifts, reported in Table **??** (Top). For instance, the hashtag *#exitpoll*, indicating the electoral outcome, becomes 10000 times more frequent on the voting day than before March 4. These shifts indicate that the frequency of hashtags reflects real-world events, thus underlining the strong link between online social dynamics and the real-world electoral process.

Closeness centrality provides different information compared to frequency: Higher closeness centrality identifies words of relevance for the cognitive processing of language through associations among concepts. In our case, associations represent co-occurrences of hashtags. In the resulting networks of hashtag co-occurrences (see also [**?**] for a similar approach) we identify hashtags with either a political or a negative connotation, see Table **??** (Bottom). The hashtag *#coalizione* becomes more central after the election day, when the electoral outcome indicate the need for a political coalition of parties. This indicates that also trends of closeness centralities reflect real-world dynamics. Through closeness centrality, we quantitatively find that negative hashtags such as *#pagliacci* and *#dimissioni* become more important after the electoral outcome, highlighting the emergence of negative feelings after the elections. A similar negative emotional dynamics was traced also in other electoral processes [**?**].

For these hashtags no significant difference was found between messages including or excluding bot accounts.

6 Testing the role of news media

In the analysis of the social bulk we identified two communities corresponding to news media accounts. In order to test for the influence of these information hubs on human-bot interactions, we disregarded all users in the above two communities identifying news media accounts. The removal of mainstream information accounts lead to negligible fluctuations (around 0.02) in the fractions of human-bot interactions (cfr. Fig. 1 (a)) and in the total volume of tweets produced by bots (around 0.4%). These results indicate that a prominent amount of human-bot interactions does not involve news media accounts and it is not influenced by the presence of information hubs.

	Frequency Increase (%)		
Top 5 Hashtag ↓	Eve	Voting	Aftermath
Elezioni4Marzo2018	$+10^4\%$	$+10^5\%$	$+10^5\%$
MaratonaMentana	$+227\%$	$+10^4\%$	$+10^4\%$
silenzioelettorale	$+10^4\%$	$+10^3\%$	-53%
exitpoll	$+270\%$	$+10^4\%$	$+10^3\%$
governopatrimoniodelpaese	$+28\%$	-92%	-97%

	Frequency Increase (%)		
Top 5 Hashtag ↓	Eve	Voting	Aftermath
italyelection	-21%	$+31\%$	$+27\%$
casta	-40%	-18%	-18%
pagliacci	-24%	$+20\%$	$+17\%$
coalizione	-18%	-15%	$+15\%$
dimissioni	-14%	-11%	$+18\%$

Table 4: Hashtags displaying the highest shifts in frequency and closeness over time. Increases are computed against the value registered in the Before time window. For instance, a frequency increase of $+10^4\%$ on Eve indicates that during the voting eve a given hashtag became ten thousand times more frequent than it was before. Frequency counts the appearance of hashtags in the observed dataset while closeness indicates how central words are in networks of semantic associations. All the considered networks contain more than $2 \cdot 10^4$ nodes so centrality estimates are not supposed to change with network size.

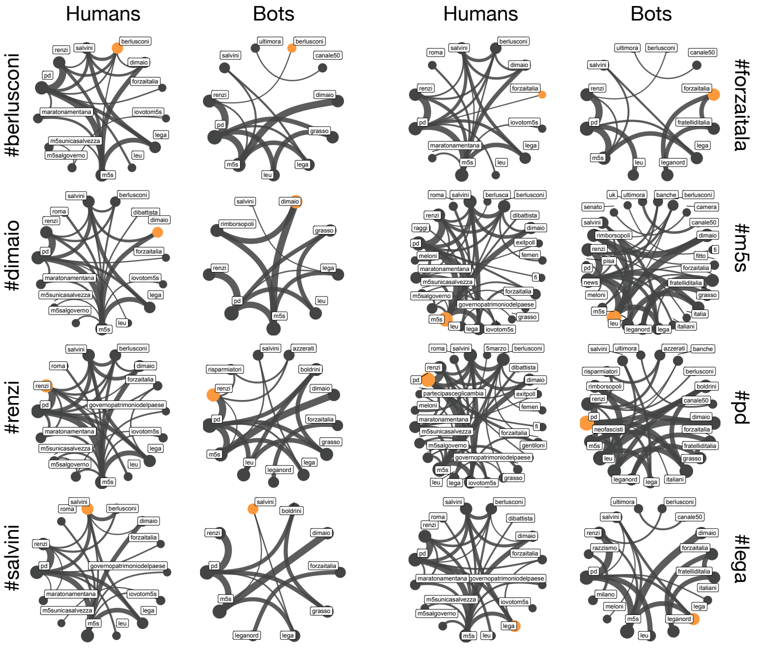

Figure 3: **Hashtags ecosystem for bots and humans.** Examples of associations of hashtags for bots and human users for the most frequent political hashtags. The considered hashtags are highlighted in orange.
